# Minute ampullary carcinoid tumor with lymph node metastases: a case report and review of literature

**DOI:** 10.1186/1477-7819-7-9

**Published:** 2009-01-22

**Authors:** Eri Senda, Koji Fujimoto, Katsuhiro Ohnishi, Akihiro Higashida, Cho Ashida, Toshio Okutani, Shigeru Sakano, Masayuki Yamamoto, Rieko Ito, Hajime Yamada

**Affiliations:** 1 Department of Gastroenterology and Hepatology, Shinko Hospital, Kobe, Hyogo 651-0072, Japan; 2 Department of Surgery, Shinko Hospital, Kobe, Hyogo 651-0072, Japan; 3 Department of Pathology, Shinko Hospital, Kobe, Hyogo 651-0072, Japan

## Abstract

**Background:**

Carcinoid tumors are usually considered to have a low degree of malignancy and show slow progression. One of the factors indicating the malignancy of these tumors is their size, and small ampullary carcinoid tumors have been sometimes treated by endoscopic resection.

**Case presentation:**

We report a case of a 63-year-old woman with a minute ampullary carcinoid tumor that was 7 mm in diameter, but was associated with 2 peripancreatic lymph node metastases. Mild elevation of liver enzymes was found at her regular medical check-up. Computed tomography (CT) revealed a markedly dilated common bile duct (CBD) and two enlarged peripancreatic lymph nodes. Endoscopy showed that the ampulla was slightly enlarged by a submucosal tumor. The biopsy specimen revealed tumor cells that showed monotonous proliferation suggestive of a carcinoid tumor. She underwent a pylorus-preserving whipple resection with lymph node dissection. The resected lesion was a small submucosal tumor (7 mm in diameter) at the ampulla, with metastasis to 2 peripancreatic lymph nodes, and it was diagnosed as a malignant carcinoid tumor.

**Conclusion:**

Recently there have been some reports of endoscopic ampullectomy for small carcinoid tumors. However, this case suggests that attention should be paid to the possibility of lymph node metastases as well as that of regional infiltration of the tumor even for minute ampullary carcinoid tumors to provide the best chance for cure.

## Background

Carcinoid tumors are generally considered to be indolent endocrine cell tumors. Ampullary carcinoid is an extremely rare tumor, and approximately 105 cases have been reported in the literature so far [1]. Whipple resection is the usual surgical treatment for this disease, but less radical procedures such as local excision or endoscopic ampullectomy have recently been reported for small carcinoid tumor [1-3], which are generally considered to be benign. Here we report a very rare case of a minute ampullary carcinoid (7 mm in diameter) that showed regional lymph node metastases, and we review the literature with emphasis on the treatment of this disease.

## Case presentation

**Figure 1:**
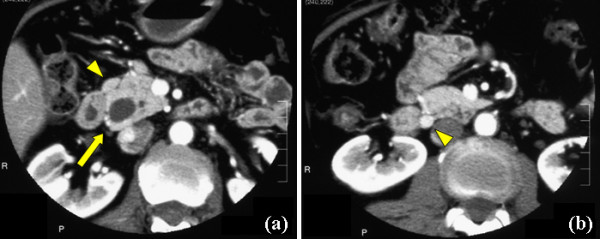
**Contrast-enhanced CT shows the markedly dilated CBD and 2 enlarged lymph nodes in the peripancreatic region**. (a) The marked dilated CBD (arrow) and one of 2 enlarged lymph nodes near the upper border of the pancreas (arrow head) are detected. (b) Another enlarged lymph node near the lower border of the pancreas (arrow head) is found.

**Figure 2:**
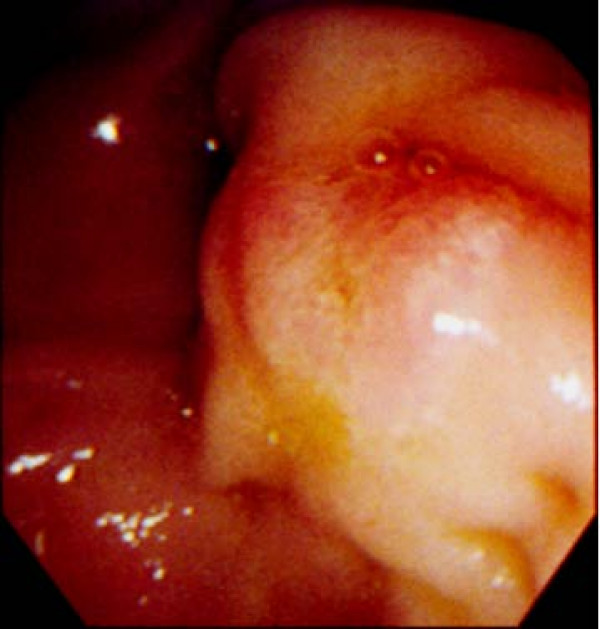
**Endoscopy shows a slightly enlarged ampullary region, suggesting the existence of a submucosal tumor because the epithelium has a normal appearance**.

**Figure 3:**
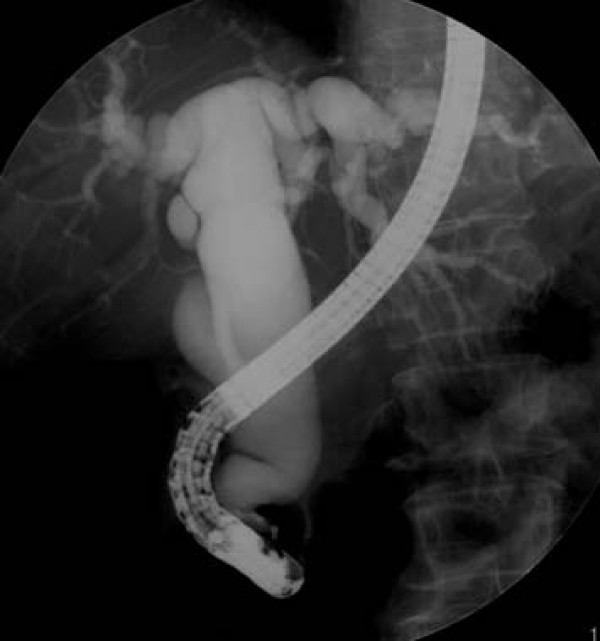
**ERCP shows severe stenosis of the distal portion of the CBD and marked proximal dilation. The main pancreatic duct is not dilated**.

The patient was a 63-year-old woman who had been attending our hospital for hypercholestelemia once a month. At her regular medical check-up, mild elevation of liver enzymes was detected, and then she was admitted to our hospital for further assessment. Contrast-enhanced computed tomography (CT) revealed marked dilatation of the common bile duct (CBD) and 2 enlarged lymph nodes in the peripancreatic region (Figure 1-a, b). Endoscopy showed that the ampulla was slightly enlarged by a submucosal tumor, although its epithelium had a normal appearance (Figure 2). Endoscopic retrograde cholangiopancreatography (ERCP) also demonstrated a markedly dilated CBD with moderate stenosis in its distal portion (Figure 3). The biopsy specimen obtained from inside the papilla after endoscopic sphinctectomy contained tumor cells with small round nuclei showing monotonous proliferation. Immunohistochemical examination demonstrated that the tumor cells were positive for neuroendocrine markers, such as chromogranin, synaptophysin, and neural cell adhesion molecule (NCAM), suggesting that the lesion was a carcinoid. Although serum serotonin and urinary 5-HIAA levels were within the normal range, a diagnosis of ampullary carcinoid tumor with local lymph node metastases was preoperatively made. She subsequently underwent the whipple resection with extended lymph node dissection. We did not perform frozen slide examination of the lymph nodes in the peripancreatic region before the resection, since the images of those enlarged lymph nodes (e.g. round shape and well-enhanced) shown by contrast-enhanced CT were typical for metastasis from carcinoid tumor as shown in Figure 1-a, b.

**Figure 4:**
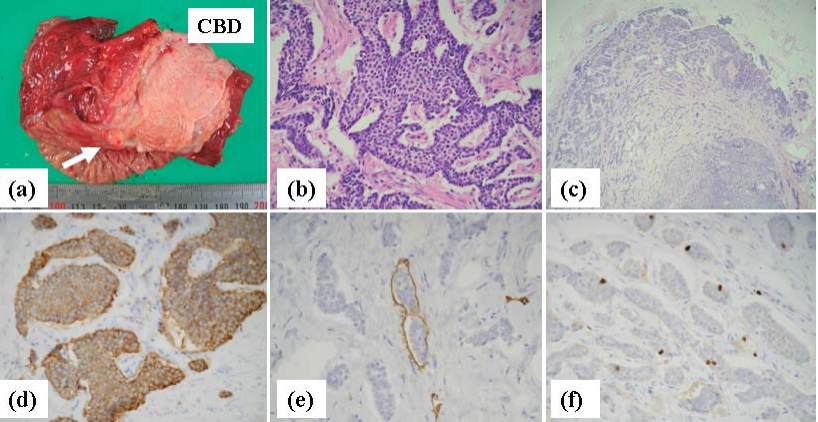
**(a) The resected specimen contains a small yellowish submucosal tumor (approximately 7 mm in diameter) located at the ampulla of Vater (arrow)**. (b) Monotonous tumor cells with small round nuclei are seen (hematoxylin and eosin staining, × 400). (c) Carcinoid tumor cells within a peripancreatic lymph node (× 200). (d) The tumor cells are positive for synaptophysin, a neuroendocrine marker (× 40). (e) Endolymphatic tumor emboli are shown by staining with D2-40 antibody (× 400). (f) Positive staining for MIB-1 antibody is seen in approximately 3.2% of the tumor cell nuclei (× 400).

The resected tumor was a small yellowish submucosal mass (7 mm in diameter) located at the ampulla of Vater (Figure 4-a). Tumor cells were detected under the ampullary epithelium, spreading over the sphincter of Oddi to reach the muscularis propria, and infiltrating into the CBD wall to create submucosal thickening (Figure 4-b). The tumor cells were also found in 2 peripancreatic lymph nodes (Figure 4-c). The tumor cells were strongly stained by synaptophysin antibody (Figure 4-d. Immunohistochemical staining using D2-40 antibody showed lymphatic involvement (Figure 4-e), and the Ki-67 labeling index of the tumor cells determined with MIB-1 was 3.2% (Figure 4-f) and overexpression of p53 was not detected. According to the classification of neuroendocrine tumors by The World Health Organization [4], our patient's tumor with regional lymph node metastases and an MIB-1 proliferative index of more than 2% was a well-differentiated endocrine carcinoma (malignant carcinoid). The patient remains free of disease and is leading a normal life at 24 months after the operation.

## Discussion

Carcinoid tumor is generally recognized to be a low-grade endocrine cell tumor derived from the endoderm. The most common site for this tumor in the digestive tract is the appendix, followed by the distal small intestine, the rectum, and the stomach [5]. Ampullary carcinoids are rare (0.05%), being even less frequent than tumors of the duodenum (2%). To date, a total of 105 cases of this tumor have been reported in the literature [5]. Jaundice (53.1%), pain (24.6%), pancreatitis (6.0%), and weight loss (3.6%) are common presenting symptoms [5,6]. Because ampullary carcinoid tends to proliferate under intact normal epithelium, this might explain the difficulty in obtaining accurate biopsy specimens by endoscopic examination and the low rate of correct preoperative diagnosis (14%) [5,7].

Many authors have suggested that Whipple resection is the best surgical option for ampullary carcinoid tumors, and the prognosis has been thought to be good with an overall survival rate of approximately 90%[7]. Meanwhile, Hwang *et al*. have recently analyzed the clinicopathological features and outcomes of 10 ampullary carcinoid patients who underwent the Whipple resection, and described that the mean tumor size was 2.1 +/- 1.3 cm and the overall survival rates were 90% at 1 year and 64% at 3 years, respectively [8]. This might suggest that this tumor is associated with a relatively poor prognosis than we think.

On the other hand, the tumors that were less than 20 mm in diameter have recently been managed by local excision [7,9], and some cases of endoscopic ampullectomy have also been reported [1-3]. Although less radical treatment strategies have been investigated to reduce surgical morbidity and preserve organ function as a reasonable alternative to pancreatic resection, there is a risk of incomplete tumor removal if preoperative evaluation is not accurate. Clements *et al. *surveyed the reports on 90 patients with ampullary carcinoid and investigated their surgical management. Twenty-two patients were treated with local excision of the tumor, which was performed on patients with tumors smaller than 20 mm in diameter. They found that one out of 22 patients died of local recurrence at 20 months after local resection [10]. Furthermore, some authors have reported that 40–50% of ampullary carcinoid tumors smaller than 20 mm in diameter were associated with metastatic disease [10,11]. Generally, it has been demonstrated that duodenal carcinoid tumors smaller than 20 mm might have a 4% incidence of metastases. These findings suggest that with respect to ampullary carcinoids, tumor size is not a reliable factor of aggressiveness.

In the present patient, 2 lymph node metastases were clearly demonstrated by CT. This finding enabled us to suspect its malignant nature preoperatively, so the Whipple procedure with regional lymph node dissection could be done. Histopathological examination revealed microscopic invasion of the lymphatics and the Ki-67 labeling index was relatively high (3.2%), even though the primary tumor was only 7 mm in diameter.

Although we also need to establish a method for identifying the extent of regional infiltration in order to determine the best treatment strategy for small ampullary carcinoids, it seems to be hard to evaluate the extent of microscopic lymphovascular invasion even if modalities such as EUS are used. Therefore, we suggest that the Whipple procedure currently remains the first choice for even small ampullary carcinoids in order to achieve complete resection of the tumor and regional lymph nodes, and that this offers the best chance of achieving a cure. Less radical endoscopic procedures should only be considered when patients have a condition that prevents the use of the Whipple procedure.

## Conclusion

Small ampullary carcinoids (less than 10 mm in diameter) are generally considered to be benign and there have been some reports of local excision or endoscopic ampullectomy for those tumors. However, we encountered the patient who had a minute ampullary carcinoid (7 mm in diameter) associated with regional lymph node metastases. This case provides evidence that carcinoid of the ampulla of Vater, irrespective of its size, might have the potential to metastasize to the regional lymph nodes, therefore, that the patients should be examined in detail concerning the existence of metastases as well as that of regional infiltration of the tumor.

## Consent

Written informed consent was obtained from the patient for publication of this case report and the accompanying images. A copy of the written consent is available for review by the Editor-in-Chief of this journal.

## Competing interests

The authors declare that they have no competing interests.

## Authors' contributions

ES drafted the case presentation and literature review sections of this manuscript. KF performed the operation, conceived of this case report, and helped to draft the manuscript. SS, MY performed the operation and postoperative management. KO, AH, CA, TO, HY carried out endoscopic examinations for the diagnosis. RI performed the pathological examination. All authors read and approved the final manuscript.
